# Periventricular Hyperintensities Mimicking Multiple Sclerosis

**DOI:** 10.7759/cureus.5326

**Published:** 2019-08-05

**Authors:** Sidra Saleem, Arsalan Anwar, Zainab Abbasi, Zauraiz Anjum, Zemal Tariq

**Affiliations:** 1 Neurology, University of Toledo, Toledo, USA; 2 Neurology, University Hospitals Cleveland Medical Center, Cleveland, USA; 3 Internal Medicine, Liaquat University of Medical and Health Sciences, Jamshoro, PAK; 4 Internal Medicine, Fatima Jinnah Medical University, Lahore, PAK; 5 Internal Medicine, Gujranwala Medical College, Gujranwala, PAK

**Keywords:** cadasil, multiple sclerosis, hyperintensity, mutation

## Abstract

Cerebral autosomal dominant arteriopathy with subcortical infarcts and leukoencephalopathy (CADASIL) is a small-to-medium-sized vessel disease that causes degeneration of vascular smooth muscles. The most frequently found mutation is NOTCH3 on chromosome 19, the presence of which confirms the diagnosis of CADASIL. The core features of CADASIL are migraine, ischemic events, cognitive decline, and psychiatric features. Its symptoms overlap with other diseases, most commonly with multiple sclerosis (MS). Both diseases can give fluid-attenuated inversion recovery in periventricular regions and deep white matter. CADASIL is often misdiagnosed and treated as MS due to these similarities. We present a case of a 28-year-old woman who began treatment for MS and was later confirmed with a diagnosis of CADASIL with a NOTCH3 mutation.

## Introduction

Cerebral autosomal dominant arteriopathy with subcortical infarcts and leukoencephalopathy (CADASIL) is a hereditary vascular disease caused by a NOTCH3 mutation, resulting in the loss and degeneration of vascular smooth muscles of small and medium arteries. The NOTCH3 mutation occurs on chromosome 19's short arm, containing 33 exons. The prevalence of CADASIL is two to four of 100,000 people [1].

CADASIL symptoms often overlap and mimic other diseases such as a sporadic disease of the small vessels, multiple sclerosis (MS), Fabry disease, and some types of leukodystrophy; of these, MS is a significant differential diagnosis [2,3]. MS is a central nervous system chronic disease characterized specifically by widespread primary demyelination, inflammation, and progressive neurodegeneration. The prevalence of MS is high, 57 to 78/100,000 in the south of the US, and 110 to 140/100,000 in the north of the US [4]. Being more prevalent, MS is often considered (incorrectly) as the first differentials in white matter lesions. We present a case that was misdiagnosed as MS and confirmed later as a rare disease, CADASIL.

## Case presentation

A 28-year-old woman presented to our tertiary care hospital reporting concerns of left arm weakness and paresthesia for three hours. The weakness started suddenly when she was sitting in her office. Two weeks before presentation, she developed right leg weakness at breakfast that resolved completely within 1.5 hours. One week before presentation, she developed the blurred vision in both eyes that also resolved completely within one hour. Her mother was diagnosed with MS at age 40 and is on treatment with frequent relapses. The rest of the patient’s medical and surgical history was unremarkable.

Her Glasgow Coma Scale score was 13/15 (eye [E] 4, verbal [V] 5, motor [M] 4). Her blood pressure was 110/70 mmHg, heart rate was 80 beats/minute, respiratory rate was 20 breaths/minute, and she was afebrile. Her neurological examination showed normal mentation with intact cranial nerves with unremarkable results from her funduscopic examination. Her motor examination showed normal bulk in all muscles with no fasciculation and tremors. Her motor strength, as tested on the Motor Research Council scale, was as follows. Her neck muscles flexion was 5/5, and extension was 5/5. Her right deltoid was 5/5, left was 4/5. Her biceps and triceps were 5/5 (right) and 3/5 (left). Her right iliopsoas was 4/5; her left was 5/5. Her knee flexion/extension was 4/5 (right), 5/5 (left), and foot dorsi/plantar flexion was 5/5. Her right biceps/triceps reflexes were 2+, her left biceps/triceps reflexes were 3+. Her patellar reflex was 2+, and her ankle reflex was 2+. Her Babinski reflex was absent. She was able to walk without support. The findings of her cerebellar and sensory exams were unremarkable.

**Figure 1:**
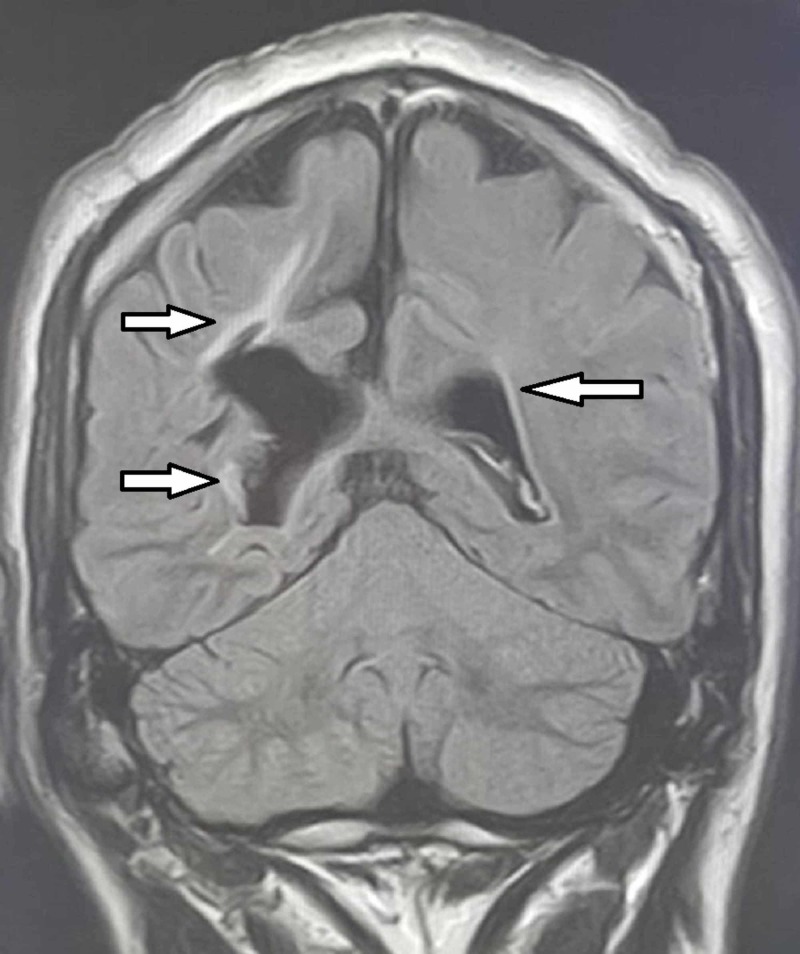
MRI sagittal section showing periventricular hyperintensities (Arrows)

Her care team suspected transient ischemic attack, and she was sent for urgent computed tomography (CT), which showed no remarkable findings. Blood and metabolic profile test findings were unremarkable, and her low-density lipoprotein was 150 mg/dl. Her cerebrospinal fluid detailed report findings were within reference ranges with no oligoclonal bands and a healthy IgG index, but her MRI showed periventricular hyperintensities in the periventricular area (Figure 1). Magnetic resonance angiography findings were unremarkable.

We started the patient on 1000 mg of IV methylprednisolone daily to treat the suspected acute flare of MS. After three days of treatment, the patient’s symptoms were improved, and she was discharged on aspirin and glatiramer acetate (GA) with instructions to return after one month for follow-up if she remains stable. She visited the outpatient clinic after six months and was doing fine, but she reported that she was experiencing difficulty concentrating and was unable to work with enthusiasm. She also reported concerns about frequent mood changes and being mostly tearful when she was formerly a very happy person. We sent the patient for a psychiatric evaluation and checked for NOTCH3 mutation. To our surprise, she had a missense mutation in exon 11 of NOTCH3, a mutation suggestive of CADASIL. Her GA was stopped, and she started aspirin and statin therapy with the advice of further cognitive evaluations and regular psychiatric assessments. She was also advised to seek genetic counseling and testing of NOTCH3 mutation in her mother.

## Discussion

Our case illustrates how CADASIL can mimic MS, which is suspected as the initial diagnosis. CADASIL is an inherited autosomal dominant disease with key features consisting of migraine, brain ischemic events, psychiatric problems including apathy and mood disorders, and cognitive issues, including dementia. These patients usually present in adulthood with variability in symptom presentation and progression. This variability is caused by environmental variables such as smoking and vascular diseases [5].

CADASIL is caused by NOTCH3 mutation. The gene has 33 exons, but 90% of mutations causing CADASIL are present between exon 2 and 24. NOTCH3 encodes a transmembrane receptor that contains epidermal growth factor-like domains; it is required for the differentiation of vascular smooth muscles. Although most interfamily mutations are the same due to environmental and epigenetic modifications, there are phenotypic variations between family members [6]. Bentley et al. also stressed that the chance of germline event (environmental or epigenetic) is accountable for phenotype variability in their research where the offspring had cord lesions, but the mother did not [7]. While heterozygous mutations are more prevalent, some cases are also reported with homozygous mutations. Ragno et al. conducted a study to determine the phenotypic distinction between heterozygous and homozygous patients of CADASIL and found that both mutations are clinically and neurophysiologically indistinguishable, emphasizing the classic definition of a dominant disease [8].

Radiological findings of CADASIL on MRI are fluid-attenuated inversion recovery involving periventricular and deep white matter, similar to MS. These periventricular lesions of MS can give classic Dawson’s fingers appearance by making an ovoid shape and radial orientation away from the ventricles, a finding characteristic of CADASIL [9,10]. Broadley et al. report that some shared NOTCH3 mutation may cause these similarities in MRI findings [10]. In exons 3 and 4, they researched the NOTCH3 gene in 745 simplex families with MS and found no proof of these mutations in patients with MS. Moreover, characteristics findings of CADASIL are hyperintense lesions in the anterior temporal pole, insular region, and external capsule. These typical features were absent in our patient, contributing to the suspicion of MS. Microscopic findings that show marked thickening of medium-sized vessels of white matter with granular deposits and increased space between the parenchyma and vessels constitute other important factors. These granular deposit stains are positive with periodic acid-Schiff (PAS) staining. The vessels of white matter also show granular osmiophilic material along the basement membrane. These combinations are characteristics of CADASIL [11].

In the literature, very few cases have been reported as CADASIL misdiagnosed as MS. A study by Sathe et al. sought to determine the number of CADASIL cases originally diagnosed and treated as MS [12]. They followed 50 CADASIL patients retrospectively by sending out a questionnaire and collecting data about initial diagnosis, onset, progression, treatment, and family history. Initially, 40% of patients were diagnosed with MS, and 16% of patients were getting an immunomodulating agent. Thirty-eight percent of patients had a positive family history of MS. Joshi et al. recorded two cases, one of which was a 23-year-old woman with scotoma and the other a 56-year-old man with the perineum and right arm paresthesia [13]. Both patients showed diffuse white matter lesions and diagnosed with MS, but subsequent NOTCH3 mutations testing confirmed both patients as having CADASIL. In his case series, O'Riordan et al. reported a similar condition where a female patient presented with an unpleasant and painful sensation of the left foot for two years [14]. That patient’s MRI showed white matter abnormalities in the external capsule and anterior temporal poles. Her NOTCH3 genetic screening was positive, and her mother, who was diagnosed with MS at age 47, was evaluated at age 62 and diagnosed with CADASIL.

CADASIL treatment focuses on symptomatic treatment of neuropsychiatric features and migraine. Secondary prevention of transient ischemic attack and stroke should be considered through statins and antiplatelet therapy. Young patients of childbearing age should be advised by a geneticist on prenatal diagnosis and pre-implantation. Genetic testing must be advised on all asymptomatic relatives of CADASIL patients [15]. This case represents another pitfall in the CADASIL diagnosis and highlights the importance of considering rare diseases when presented with patients with white matter lesions.

## Conclusions

CADASIL is a small vessel disease that can present with different phenotypes. These phenotypes and radiologic findings of hyperintensities can mimic other diagnoses, particularly MS. Testing these patients with NOTCH3 mutation is always important to include or exclude CADASIL and prevent the delay in diagnosis as it is essential for patients and their family members to receive genetic counseling.
